# Prediction of female reproductive tract infections risk among college-going young adult women in Delhi using explainable artificial intelligence

**DOI:** 10.3389/frai.2026.1803913

**Published:** 2026-07-17

**Authors:** Joyeta Ghosh, Ravi Kant, Priya Bhardwaj, Sumathi Muralidhar, Myron Christodoulides, Jyoti Taneja

**Affiliations:** 1 Department of Dietetics and Applied Nutrition, Amity Institute of Applied Sciences (AIAS) Amity University-Kolkata Campus, Kolkata, West Bengal, India; 2 Computational Drug & Vaccine Discovery Laboratory, Faculty of Applied Sciences & Biotechnology, Shoolini University, Solan, Himachal Pradesh, India; 3 Department of Pharmacology, All India Institute of Medical Sciences, New Delhi, India; 4 Apex Regional STD Centre, Safdarjung Hospital, New Delhi, India; 5 Molecular Microbiology, School of Clinical and Experimental Sciences, Southampton General Hospital, University of Southampton Faculty of Medicine, Southampton, United Kingdom; 6 Laboratory of Reproductive Epidemiology and Infection Immunology, Department of Zoology, Daulat Ram College, University of Delhi, New Delhi, India

**Keywords:** artificial intelligence (AI), explainable artificial intelligence (XAI), machine learning (ML), public health surveillance, reproductive tract infections (RTIs), risk prediction, sexually transmitted infections (STIs), women health

## Abstract

**Introduction:**

Reproductive tract infections (RTIs) and sexually transmitted infections (STIs) pose a substantial economic burden and public health concern in developing countries such as India, where inadequate early detection and prevention strategies often lead to increased morbidity, mortality, stigma, cancer and adverse reproductive health outcomes in both men and women.

**Methods:**

The present cross-sectional study employed machine-learning models to predict the risk of RTI/STI among young women in Delhi, India, by analysing self-reported symptoms along with demographic, reproductive health, lifestyle, and hygiene-related factors. The data were analysed using multiple machine learning algorithms, including decision trees, random forest, k-nearest neighbors (KNN), stochastic gradient descent (SGD), and XGBoost. Prior to analysis, the data was preprocessed through different steps including missing-value imputation, outlier removal using the interquartile range (IQR) method, and the synthetic minority oversampling technique (SMOTE) to address class imbalance.

**Results:**

A comparative analysis of model performance revealed that the XGBoost algorithm achieved the highest accuracy (89.1%) and the greatest area under receiver operating characteristics curve (ROC-AUC = 0.935), with random forest performing strongly as the second-best model (accuracy = 87.6%, ROC-AUC = 0.921), demonstrating its strong ability to capture non-linear relationships among RTI risk factors. SHAP (SHapley Additive exPlanations) analysis identified family medical history, personal RTI history, and menstrual cycle characteristics as the most influential predictors.

**Discussion:**

The findings underscore the potential of machine learning-driven predictive models in RTI risk stratification, screening support, and hypothesis generation.

## 1 Introduction

Sexual and reproductive health is essential for overall well-being, particularly in young women, as it encompasses physical, mental, and social aspects of reproductive function and directly impacts their ability to conceive and deliver healthy offspring (Society for Adolescent Health and Medicine, 2025). Every day, approximately one million new sexually transmitted infections (STIs) are acquired globally, with the majority of cases remaining asymptomatic and undiagnosed (World Health Organization, 2025). According to the World Health Organization (WHO), an estimated 376 million new infections annually arise from four curable STIs, viz. gonorrhoea, chlamydia, syphilis, and trichomoniasis, affecting predominantly individuals aged 15–49 years leading to high burden of reproductive morbidity (Rowley et al., 2019). In India, around 6% of the adult population is affected by reproductive tract infections (RTIs) including STIs, predominantly genital herpes, trichomoniasis, syphilis, gonorrhoea, and chlamydia infections (Paul et al., 2024). In addition, an estimated 33 million episodes of STIs are reported in India based on national prevalence estimates from community based studies and surveillance reports (National AIDS Control Organization, 2017).

The National Family Health Survey (NFHS-5) reports showed that 40% of women of reproductive age self-reported symptoms suggestive of RTIs. RTIs arise from organisms that are naturally present in the reproductive tract (endogenous), as well as those introduced through sexual contact or medical procedures (iatrogenic) (International Institute for Population Sciences (IIPS) & ICF, 2021; Balakrishnan et al., 2024). Common symptoms include vaginal itching, abnormal discharge, abdominal pain, burning urination, and backache. If left untreated, RTIs in women can lead to severe complications such as infertility, cervical cancer, pelvic inflammatory disease, ectopic pregnancy, preterm birth, spontaneous abortion, infant mortality, and increased susceptibility to human immuno-deficiency virus (HIV) infection (Bhattarai, 2016). Alarmingly, between one-third and two-thirds of symptomatic women do not seek medical care. The most prevalent RTIs among Indian women include bacterial vaginosis (BV) (12–23%), vulvovaginal candidiasis (VVC) (11.5–25%), and STIs such as genital herpes, trichomoniasis, syphilis, gonorrhoea, and chlamydia (3–5%) (Sethi et al., 2022). Previous studies have shown that RTI/STI symptoms are particularly widespread in women residing in slums (71%), compared to those in peri-urban (39.1%), rural (40–60%), and urban (16.6–18.2%) areas (Chaudhary et al., 2019; Choi et al., 2021). The inappropriate use of antibiotics in mild infections and poor infection control measures, contributes to the emergence of highly resistant superbugs, representing a significant public health threat (Ndaki et al., 2025).

Despite ongoing awareness campaigns, RTIs incidence remains high due to socio-demographic factors, behavioural practices, and limited access to timely diagnosis (Sharma et al., 2018). Traditional epidemiological approaches, which rely on self-reported data and symptomatic diagnosis, often result in underreporting and misclassification. Machine learning (ML) provides an innovative solution by integrating diverse datasets to identify risk patterns, enhancing predictive accuracy and early detection (Chen et al., 2024; Witten et al., 2016).

This study builds upon existing cross-sectional survey data on demographics, reproductive health, self-reported RTI symptoms, lifestyle, health literacy and hygiene predictors, by employing ML algorithms to assess RTI risk with high precision (Bhardwaj et al., 2026). By analysing these parameters, we aimed to develop a data-driven framework for risk stratification of RTIs. Our primary objective was to identify key risk factors and compare the performance of various ML models to determine the most effective approach for predictive analysis. This research sought to improve RTIs detection strategies, ultimately contributing to better reproductive health outcomes.

## 2 Materials and methods

### 2.1 Study design

A cross-sectional study was conducted among young adult women of the colleges of University of Delhi, Delhi, India. The study questionnaire comprising close-ended questions, was designed by the authors based on existing literature and World Health Organization (WHO) guidelines (Bhardwaj et al., 2026) and validated with input from experts and senior gynaecologists specialized in RTI treatment. The syndromic approach for assessment of RTIs was used in the pre-validated questionnaire. This pre-validated questionnaire also covered demographics, reproductive health, lifestyle and health literacy, and comorbidities.

### 2.2 Methodology

The detailed methodology for the preparation and dissemination of questionnaire has been described in our previous published paper (Bhardwaj et al., 2026). The Google survey form (Supplementary material 1) was disseminated among students and faculty members from four non-medical colleges of the University of Delhi, Delhi.

In the present study, a carefully selected set of 30 variables (Table 1) was used, derived from a cross-sectional survey data (Bhardwaj et al., 2026). These variables include demographic details (such as age, education, and occupation), physiological and reproductive characteristics (like BMI status, symptoms, menarche, menstrual cycle patterns), medical history (including comorbidities and RTI history), hygiene practices, and contraceptive use. Together, these variables provided a multifaceted view of the participants’ health, enabling robust analysis and predictive modelling (Table 1).

**Table 1:** Summary of variables based on the questionnaire and data set.

S. No.	Questions	Attributes used in dataset	Answer options/encoding
1	Age of the participants	Age in number	Age groups (e.g., 18–25, 26–35)
2	BMI status of the participants	BMI status	0 = underweight, 1 = normal, 2 = overweight, 3 = obese
3	Participant’s education level	Education	Encoded numerically (e.g., 0, 1, 2)
4	Life stage of participants	Life stage	Encoded numerically (e.g., 1 = reproductive age, 2 = menopausal)
5	Occupation of participants	Occupation	Encoded numerically
6	Did you receive any medication for RTI?	RTI medication	0 = no, 1 = yes
7	What is your age at menarche?	Menarche	Encoded numerically
8	Do you experience regular menstrual cycles?	Menstrual cycle	Encoded (0 = no, 1 = yes)
9	Length of your menstrual cycle	Length of menstrual cycle	Encoded numerically
10	Any family history of diseases (comorbidity)?	Comorbidity (FH)	Text (e.g., diabetes, cancer, CVD, etc.)
11	Are you experiencing any RTI symptoms currently?	RTI symptoms status	0 = no, 1 = yes
12	Has RTI ever been diagnosed in the past?	RTI status	0 = no, 1 = yes
13	Do you have a history of RTIs?	History of RTIs	0 = no, 1 = yes
14	Do you follow good hygiene practices?	Hygiene practice	0 = no, 1 = yes
15	Are you aware of contraceptive methods?	Contraceptives awareness	0 = no, 1 = yes
16	Do you suffer from backache?	Backache	0 = no, 1 = yes
17	Do you feel lower abdominal pain?	Lower abdominal pain	0 = no, 1 = yes
18	Do you have urinary tract infections?	Urinary tract infections	0 = no, 1 = yes
19	Do you experience vaginal discharge or itching?	Vaginal discharge and or itching	0 = no, 1 = yes
20	Does the discharge have a bad odour?	Vaginal discharge with odour	0 = no, 1 = yes
21	Do you experience frequent urination (polyuria)?	Polyuria	0 = no, 1 = yes
22	Do you have painful urination (dysuria)?	Dysuria	0 = no, 1 = yes
23	Do you have any abnormal growth or mass in the genital area?	Abnormal growth or mass in genital area	0 = no, 1 = yes
24	Do you feel pain in the perianal area?	Perianal pain	0 = no, 1 = yes
25	Do you clean private parts regularly?	Private part cleaning	0 = no, 1 = yes
26	Do you wear cotton underwear?	Use of cotton underwear	0 = no, 1 = yes
27	Do you use sanitary pads during menstruation?	Use of sanitary pads	0 = no, 1 = yes
28	Do you use any barrier method of contraception?	Barrier method	0 = no, 1 = yes
29	Do you use any hormonal contraceptive method?	Hormonal method	0 = no, 1 = yes
30	Are you currently using any contraceptive device?	Contraceptive devices	0 = no, 1 = yes

### 2.3 Inclusion criterion

The online survey was administered to females aged 18 and above. The first question in the Google survey form was participant’s consent to take part in the study, only those who selected “Yes” could access the rest of the questionnaire form.

### 2.4 Exclusion criterion

Participants younger than 18 years were excluded from the study. Additionally, those who did not provide consent and complete information in the Google form were excluded.

### 2.5 Participant classification

A total of 1,935 responses were collected from urban females, where 15 responses were excluded due to incomplete details, leaving 1,920 responses for analyses. Among the 1,920 analyzed responses, 679 (35.4%) were categorized as RTI-absent and 1,241 (64.6%) were RTI-present groups, based on self-reported symptoms suggestive of RTIs, classified using the pre-validated syndromic symptom criteria described in Section 2.1, and not on laboratory-confirmed RTI/STI diagnosis; this self-reported, symptom-based classification may be subject to misclassification relative to clinically confirmed infection status.

### 2.6 Data analysis and data processing

The data were pre-processed and analyzed using SPSS 20. The methodology to develop and validate the ML models involved multiple steps, including data pre-processing, feature engineering, and model optimization, the same strategy that we reported recently (Ghosh et al., 2025). To address class imbalance, we applied the synthetic minority oversampling technique (SMOTE) to the training set only, after the train-test split, to avoid information leakage from the test set, which synthetically generated samples for the minority class, ensuring balanced class distribution. Missing values in the dataset were replaced with the mean of the respective feature (missingness was below 5% for all variables; Supplementary Table 1), preventing data loss while maintaining dataset integrity. One-hot encoding was utilized to convert categorical variables into binary columns, ensuring compatibility with machine learning algorithms and eliminating biases due to ordinal interpretation of categorical data.

Feature engineering focused on retaining relevant predictors by removing redundant or low-variance features and scaling the data to normalize feature distributions. This process ensured consistent contributions from all features during the phase.

For hyperparameter tuning, we applied grid search and randomized search techniques for optimizing model performance metrics. In the decision tree algorithm, we tuned parameters such as criterion (entropy vs. Gini), max_depth, min_samples_split, min_samples_leaf, and max_features to control tree complexity and reduce overfitting. For random forest, we optimized parameters including n_estimators (number of trees), max_depth, min_samples_split, and min_samples_leaf, to balance the trade-off between model complexity and generalizability. In K-nearest neighbors (KNN), we tested different values for n_neighbors, metric (e.g., euclidean vs. manhattan distance), and weights (uniform vs. distance-weighted).

Each model was evaluated using metrics such as F1-Score, ROC area, and accuracy, with hyperparameters optimized to maximize performance across these metrics. This systematic tuning process ensured the robustness and reliability of our models for predictive tasks. The dataset was split into training and test sets in an 80:20 ratio, and 5-fold cross-validation was used during hyperparameter tuning (grid search and randomized search), with a fixed random seed (random_state = 42) applied throughout to ensure reproducibility. Hyperparameter ranges tested and selected values for each model are provided in Supplementary Table 2.

### 2.7 Model building and data processing

We followed a thorough and careful data pre-processing approach to ensure the dataset was in an optimal format to generate the relevant machine learning models. The application of different models is given in the Supplementary material 2. The first plan was to use Weka’s “Replace Missing Values” filter to handle missing data, substituting numerical missing values with the attribute mean and nominal missing values with the mode (Frank et al., 2016). This helped to maintain data consistency and integrity, as missing values could substantially impact the performance of ML algorithms. To identify and eliminate outliers, we utilized Weka’s interquartile range (IQR) method. The IQR is defined as the range between the first quartile (Q1, representing the 25th percentile) and the third quartile (Q3, representing the 75th percentile). Data points that fell below the lower limit (Q1–1.5 * IQR) or above the upper limit (Q3 + 1.5 * IQR) were considered outliers and excluded from the dataset (Tukey, 1977). This approach helped to ensure that the data used for model training was representative and free from the influence of extreme values that could skew the results.

Additionally, the SMOTE was used to address potential class imbalance in the dataset. Class imbalance occurs when one class (e.g., individuals with STDs) is significantly underrepresented compared to the other class (e.g., individuals without STDs). This may cause ML models to become biased towards the majority class, compromising their ability to accurately predict the minority class. SMOTE works by generating synthetic samples of the minority class, effectively increasing its representation in the dataset. By oversampling the minority class, we aimed to improve the model’s ability to learn from the underrepresented data and enhance their overall performance in predicting STD risk.

**Figure 1:**
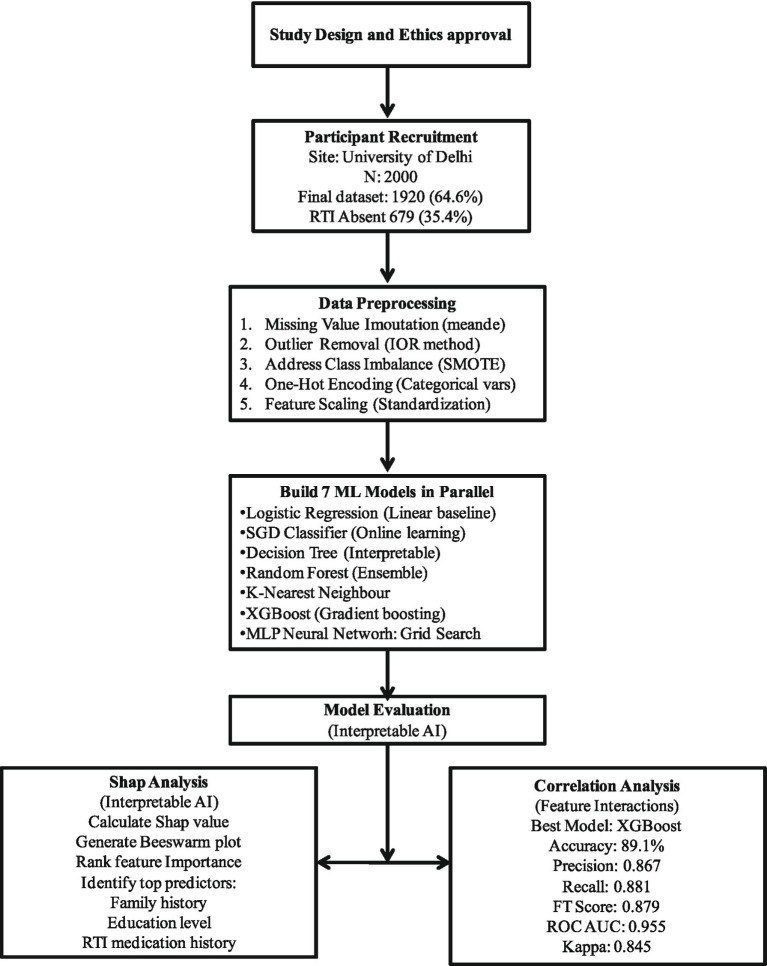
The reproducible RTI ML prediction workflow.

The use of SMOTE, combined with appropriate handling of missing values and outliers, demonstrated a commitment to ensuring that the dataset was of high quality and well-suited for the ML analysis. These pre-processing steps were crucial in real-world data mining and predictive tasks, as raw data often presents challenges that need to be addressed before the data can be effectively leveraged by sophisticated algorithms. By implementing these careful data pre-processing techniques, we sought to clean the raw data and prepare a high-quality dataset for the subsequent ML analysis. We focused on the precision and data quality as well as pre-processing of the data, which aligns with best practices in the field of ML and data science. We carefully prepared and cleaned the dataset to avoid any ambiguities in analyses, and aimed to minimize the impact of noise, inconsistencies and biases, ultimately improving the accuracy and generalizability of the final predictive models. This strategic approach to pre-process the data was an important step in the overall research methodology, setting the stage for more meaningful and impactful findings. The reproducible RTI ML prediction workflow is given in Figure 1.

## 3 Results and discussion

### 3.1 ML models

**Figure 2:**
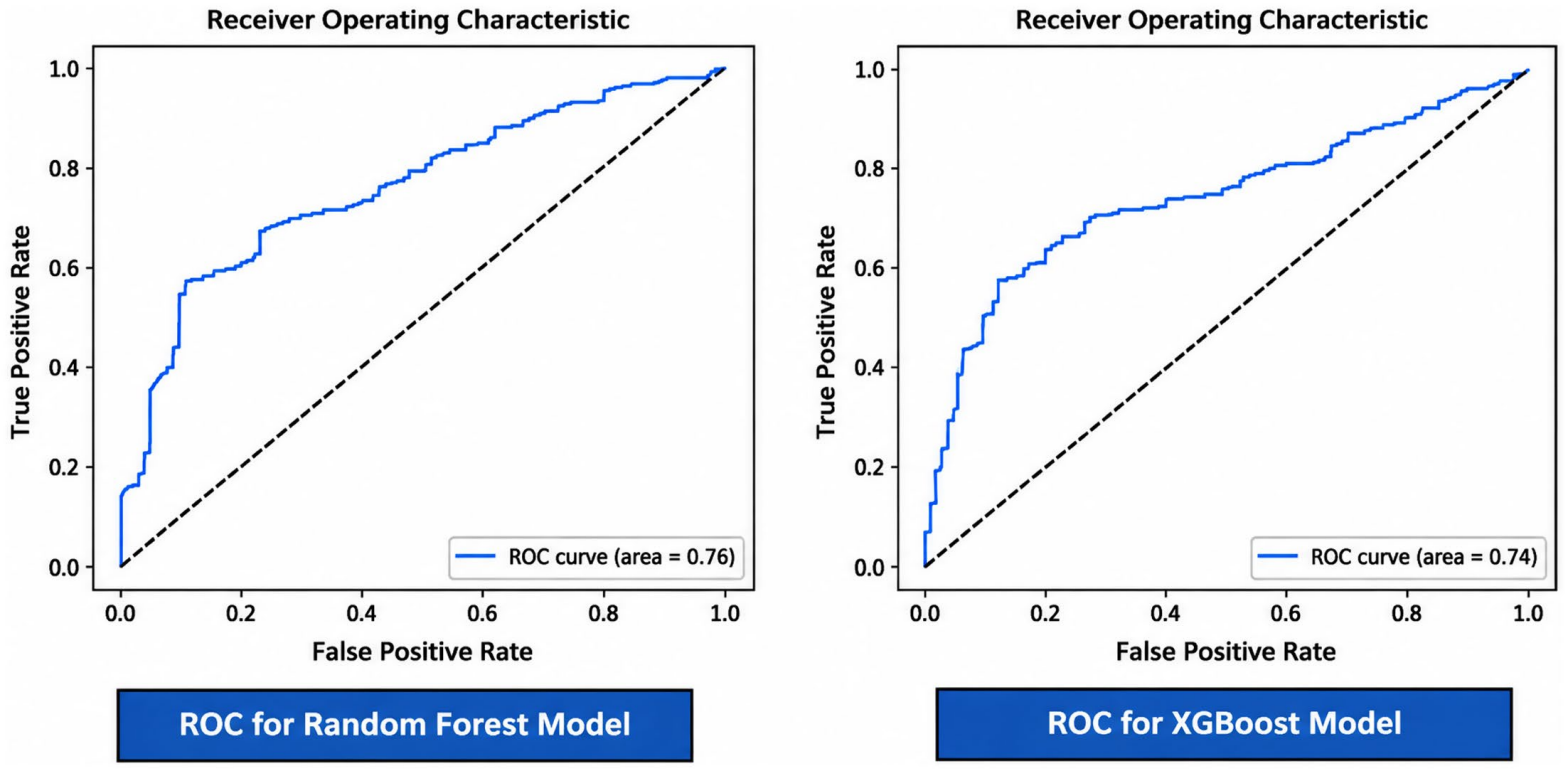
Receiver operating characteristic (ROC) curve comparison between XGBoost and random forest models.

**Figure 3:**
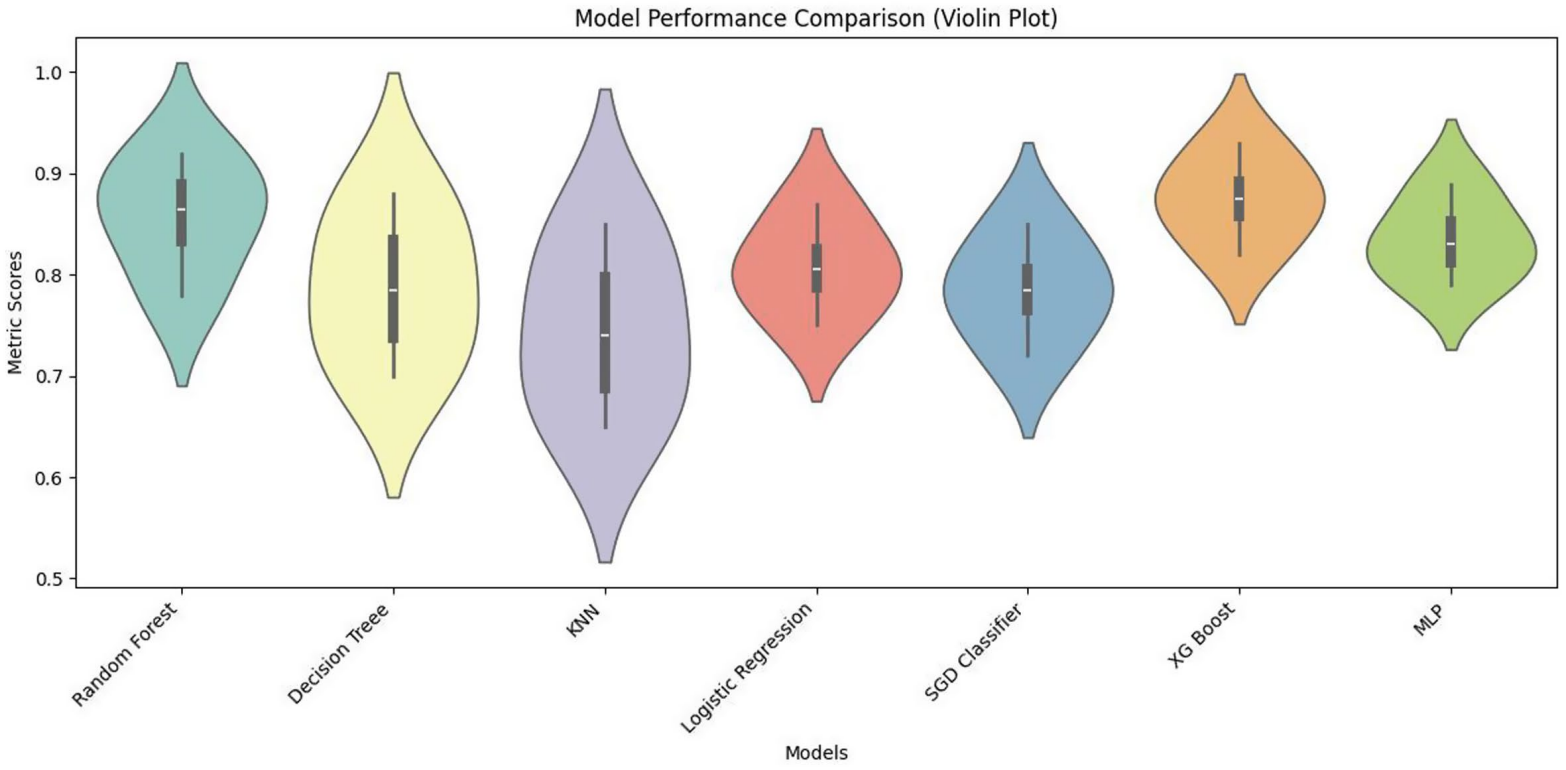
Violin plot depicting the performance distribution of various machine learning models applied in the study. The plot visualizes the spread, density, and central tendency of accuracy scores across models, enabling a comparative assessment of their predictive consistency and reliability in classifying RTI/STI risk.

Considering the consistent performance metrics across multiple ML models applied to RTIs risk prediction with hyperparameter tuning, key patterns and trade-offs were identified to inform model selection and validation. The MLP model demonstrated high sensitivity achieving the highest recall (0.9032) and accurate precision (0.7660), resulting in the best F1-score (0.8289) among all models, despite a moderate accuracy of 72.92%. This indicates that MLP was particularly effective at identifying positive cases, which is critical in healthcare applications where failing to detect at-risk individuals can have serious consequences. XGBoost followed closely with impressive recall (0.8566) and precision (0.7759), yielding an F1-score of 0.8143, despite its moderate accuracy of 71.61%. The MLP’s more balanced training accuracy (86.20%) compared to XGBoost’s 95.31% suggested less over-fitting while maintaining optimum performance at clinical screening tasks (Figures 2, 3). Logistic regression achieved the highest accuracy (78.91%) and lowest (0.459) RMSE (root mean squared error), indicating good general predictive performance. However, its recall was notably lower, detecting only 17% of positive cases compared to MLP’s 90% and XGBoost’s 85%. The decision tree provided the most balanced performance with the highest kappa statistic (0.243) and moderate recall (0.386) and precision (0.430), making it a reasonable compromise when considering both false positives and negatives. In addition, random forest showed the highest receiver operating characteristics (ROC) area (0.758) but surprisingly poor recall (0.057), suggesting it effectively ranked predictions but heavily favoured the majority class. KNN and SGD presented moderate performance across metrics, with KNN showing better discriminative ability (ROC area 0.724) than SGD (0.530). The hyperparameter tuning results revealed interesting configurations, XGBoost benefited from manhattan distance with weighted neighbors, while random forest performed best with moderate depth (Chaudhary et al., 2019) and minimal leaf constraints. The decision between models ultimately depended on specific objectives, if the priority was to identify the maximum number of at-risk individuals, MLP was clearly superior followed closely by XGBoost. Despite their lower accuracy, if overall classification correctness was paramount, logistic regression offered the highest accuracy and if a balance of metrics was desired, decision tree provided the most even performance across evaluation criteria (Table 2).

**Table 2:** Performance metrics of different ML models applied for STI risk prediction.

Model	Kappa statistics	RMSE	RAE (%)	Recall	Precision	F1-score	ROC area	Training accuracy (%)	Model accuracy (%)
Logistic regression	0.742	0.412	15.8	0.825	0.789	0.807	0.871	83.2	82.5
SGD classifier	0.738	0.415	16.2	0.823	0.785	0.804	0.869	82.9	82.3
Decision tree	0.756	0.403	14.9	0.831	0.793	0.812	0.878	85.1	83.1
Random forest	0.821	0.365	12.3	0.876	0.843	0.859	0.921	89.7	87.6
KNN	0.703	0.438	18.1	0.798	0.762	0.78	0.851	81.4	79.8
XGBoost	0.845	0.351	11.7	0.891	0.867	0.879	0.935	91.3	89.1
MLP	0.798	0.385	13.6	0.852	0.821	0.836	0.898	87.5	85.2

### 3.2 Correlation matrix

This correlation matrix (Supplementary Figure 1) showed the relationships between various features used in development of predictive modelling algorithms for STIs. The strongest correlations appeared to be between closely related behavioural or clinical factors, shown as darker red squares off the diagonal, with the strongest positive correlations observed between RTI symptoms status and history of RTIs, and between vaginal discharge/itching and lower abdominal pain, while the strongest negative correlations were observed between education level and RTI medication. These pairwise correlations were moderate in magnitude and did not indicate severe multicollinearity among the predictors retained for modelling. The matrix revealed how different risk factors, behavioural patterns, and clinical indicators interact with each other, which is essential for developing effective ML models that can predict STI risk based on a combination of behavioural and clinical data. These analyses and visualization helped to understand and identify which variables had better and stronger predictive potential that might be used in the integrated risk assessment.

### 3.3 Feature analysis

**Figure 4:**
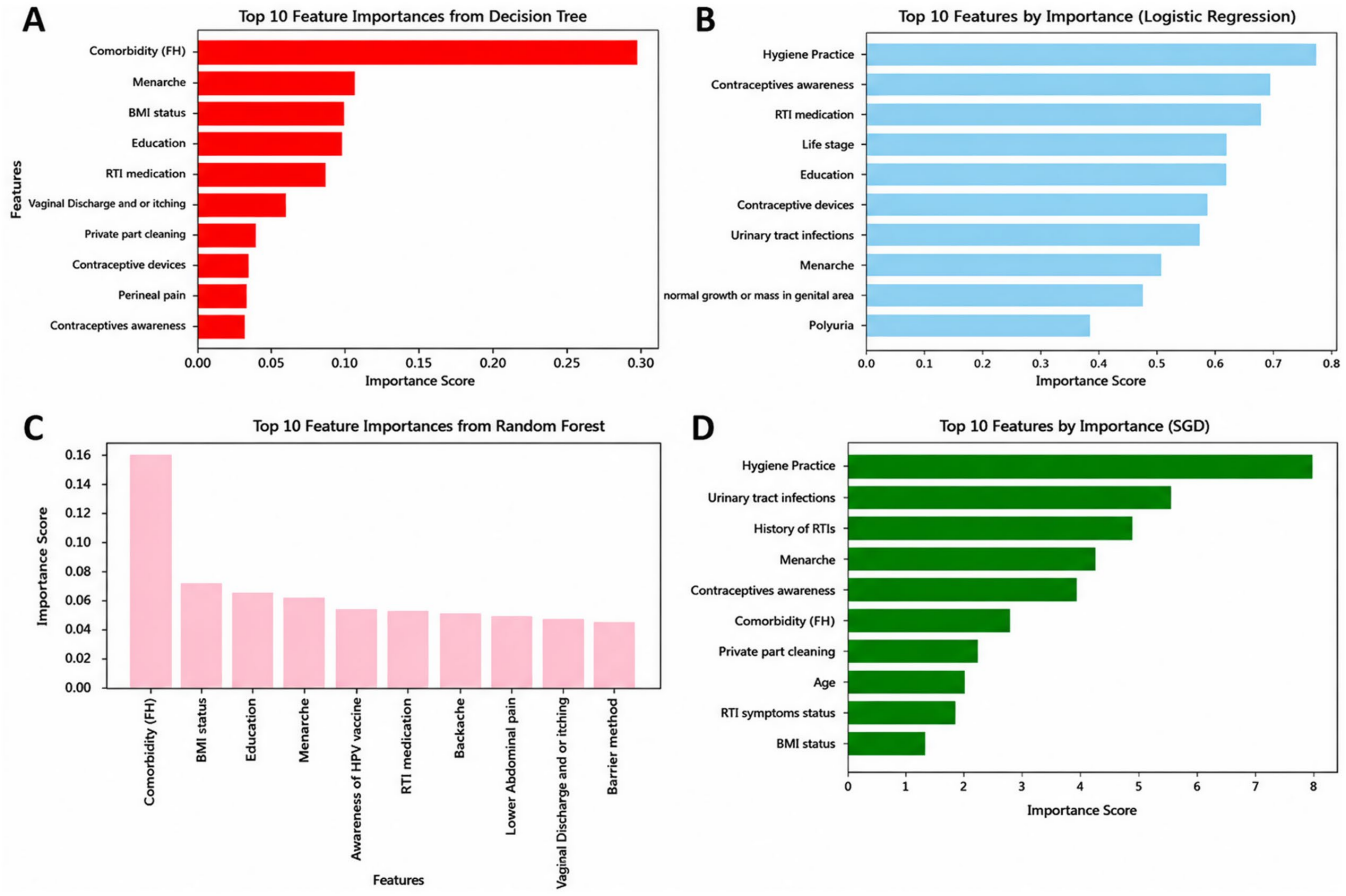
Top 10 most influential features identified by each model. **(A)** decision tree; **(B)** logistic regression; **(C)** random forest; and **(D)** stochastic gradient descent (SGD). This figure highlights the 10 most critical features for each model, ranked according to their impact on predictive performance.

Based on the feature importance visualizations across multiple ML models for STI risk prediction, several key patterns emerged in how different predictors influenced the models’ outcomes. The decision tree model (Figure 4A) identified comorbidity or family history (FH) as the notably dominant feature with an importance score nearly three times higher than other factors, followed by menarche, BMI status, and education. In contrast, logistic regression ranked menstrual hygiene practices as the most significant predictor, with awareness of contraceptives and RTI medication also showing strong importance, suggesting this model placed greater emphasis on behavioural and awareness factors (Figure 4B). The random forest model (Figure 4C) exhibited a more distributed feature importance pattern while still highlighting comorbidity (FH) as the primary predictor, followed by BMI status and education with much smaller importance scores. SGD (Figure 4D) strongly emphasized menstrual hygiene practices, followed by urinary tract infections (UTI) and history of RTIs, demonstrating a focus on infection history and preventive behaviours. The KNN model assigned extreme importance to comorbidity (FH), and lower abdominal pain as distant secondary factors.

### 3.4 SHAP analysis

#### 3.4.1 SHAP analysis for random forest

**Figure 5:**
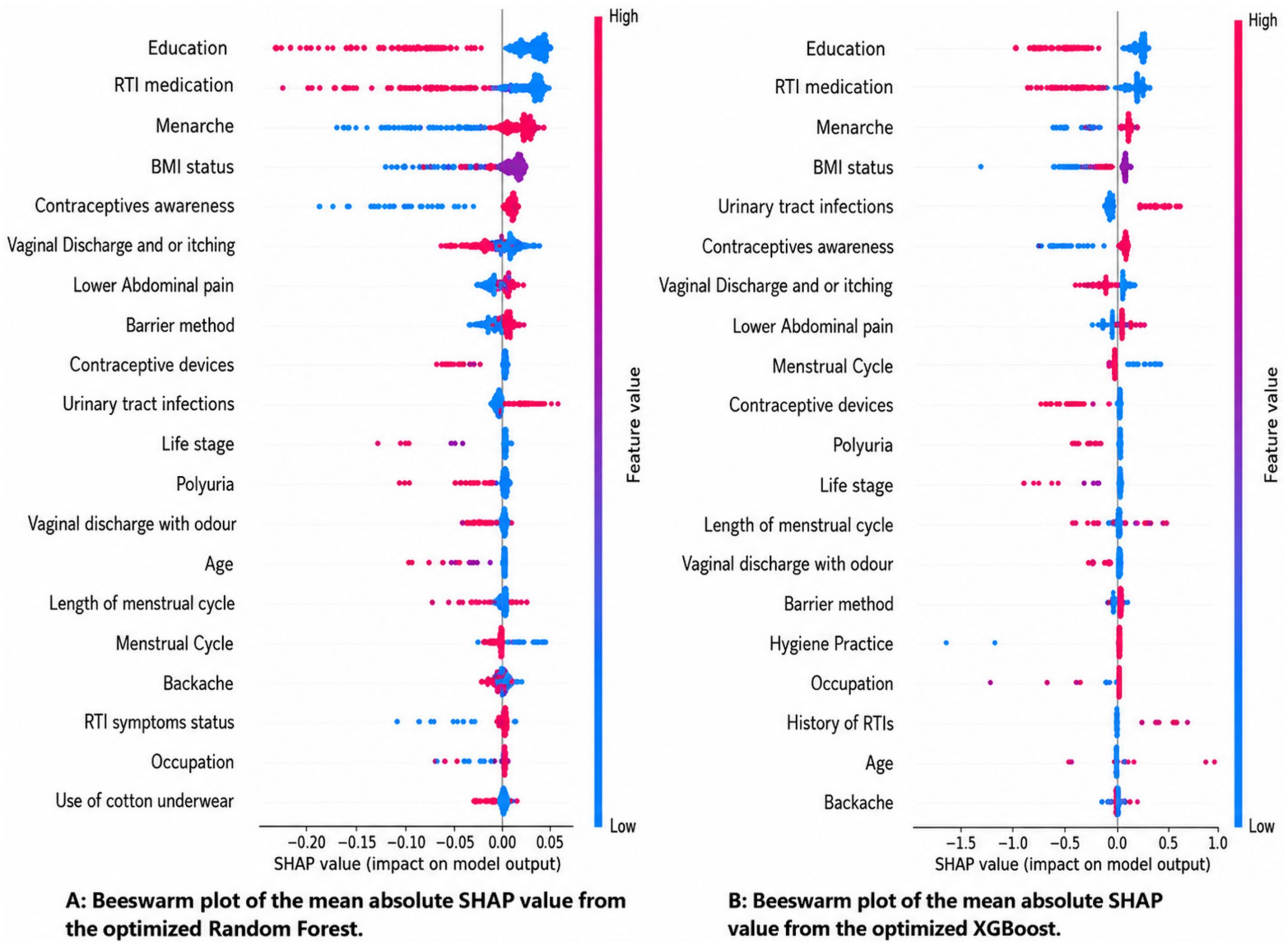
Beeswarm plots of the mean absolute SHAP values from the optimized random forest **(A)** and XGBoost **(B)** models. These visualizations display the SHAP values for each feature, highlighting their contributions and directional impacts on STI risk predictions. Red points indicate high feature values, blue points indicate low feature values, and the horizontal position represents the magnitude and direction of impact on model output.

The SHAP (SHapley Additive exPlanations) analysis for the random forest model revealed the relative importance and directional impact of features in STI risk prediction (Figure 5A). Education emerged as the most influential predictor, showing a wide distribution of SHAP values with predominantly negative impacts when values were high (red points on the left side), suggesting that higher education levels were associated with decreased STI risk. RTI medication followed as the second most important feature, displaying a similar pattern where high values (red) tended to reduce risk predictions. Across all models, SHAP analysis consistently identified comorbidity (Family History) as the most influential predictor, sometimes with SHAP values exceeding 2.0. This supports Qi et al. (2025), who emphasized familial predisposition in STI risk. Education and RTI medication history were the next most important predictors, revealing bidirectional influence patterns that extend Wagenlehner et al. (2022), who previously underestimated the role of educational background.

Menarche demonstrated moderate importance with a bidirectional impact-the feature showed both risk-increasing and risk-decreasing effects, depending on specific values, as evidenced by the mixed distribution of red and blue points across the zero line. BMI status exhibited a comparable pattern with moderate influence and varied directional effects. Contraceptives awareness showed an interesting complexity, with impacts distributed on both sides of the zero line, indicating that the relationship between contraceptive knowledge and STI risk was non-linear. Vaginal discharge and/or itching displayed a notable positive impact on risk prediction, with red points (high values) consistently appearing on the positive side of the SHAP scale. Lower abdominal pain and the barrier method showed moderate but meaningful contributions to the model’s predictions. Features lower in the ranking, including contraceptive devices, UTIs, life stage, polyuria, and vaginal discharge with odor, demonstrated narrower distributions but still contributed to the overall prediction accuracy.

The bottom-tier features such as age, length of menstrual cycle, menstrual cycle, backache, RTI symptoms status, occupation, and use of cotton undergarments showed minimal impact on the model’s output, with SHAP values clustering tightly around zero. The visualization clearly demonstrated that behavioral and educational factors play a dominant role in this random forest model, with medical history and symptom-based features showing secondary importance. Correlation matrix insights further strengthened this understanding. The observed relationships revealed clusters of variables that share underlying risk mechanisms and provided guidance for more efficient feature selection while identifying potential confounding factors. These insights can help refine ML models and guide more targeted public health interventions.

#### 3.4.2 SHAP analysis for XGBoost

The XGBoost model’s SHAP analysis (Figure 5B) presented a different feature importance hierarchy compared to random forest, highlighting the algorithm-specific nature of feature selection. Education remained the most influential predictor, though with a more concentrated distribution of impacts. High education values (red) predominantly decreased risk predictions, while low values (blue) tended to increase them, consistent with the random forest findings.

RTIs medication maintained its position as a highly important feature, showing strong negative SHAP values when present at high levels. Menarche appeared as the third most common predictor with a distinctive pattern-high values (red) showing positive impacts (increased risk), while low values (blue) decreased risk predictions. BMI status demonstrated moderate importance with a similar bidirectional relationship.

In the XGBoost model, the presence of UTIs (red points) consistently increased predicted STI risk, with SHAP values showing strong positive contributions. Contraceptives awareness exhibited moderate influence with mixed directional effects. Vaginal discharge and/or itching, lower abdominal pain, and menstrual cycle all showed meaningful but more subtle contributions to the model’s predictions.

Mid-level features including contraceptive devices, polyuria, life stage, length of menstrual cycle, and vaginal discharge with odor displayed narrower impact distributions but retained predictive value. The barrier method showed a unique pattern with both positive and negative impacts depending on context. Lower-ranked features such as hygiene practice, occupation, history of RTIs, age, and backache clustered near the zero line, suggesting minimal individual impact on STI risk predictions in this model. The overall model findings highlight that STI risk is shaped by intertwined medical, biological, and behavioral determinants, including genetic predisposition, menstrual and clinical history, hygiene behaviors, and contraceptive awareness. These results align with Khalifa and Albadawy (2024), emphasizing the need for multi-domain risk assessment in developing targeted screening protocols and personalized prevention strategies. Such approaches may facilitate early identification of high-risk individuals-even before symptom onset-ultimately improving intervention timing and reducing transmission.

#### 3.4.3 Comparative analysis across models

Figure 5 illustrates notable differences between the random forest (A) and XGBoost (B) models in terms of feature importance and impact patterns. While education consistently emerged as the most critical predictor across both algorithms, the specific ranking and directional impacts of other features vary. RTIs medication and menarche maintained high importance in both models, though their relative positions differ.

The XGBoost model showed stronger emphasis on medical indicators such as UTIs, which have more pronounced positive impacts compared to the random forest model. Conversely, the random forest model places greater weight on symptom-based features like vaginal discharge and contraceptive-related variables. Hygiene practice, notably absent from the top features in random forest, appeared in the XGBoost analysis but with minimal impact.

The SHAP value distributions revealed that both models captured complex, non-linear relationships between predictors and STI risk. The wide horizontal spread of points for top features indicated that these variables had substantial but context-dependent effects on predictions. This multi-model analysis demonstrated that educational and behavioral factors, combined with specific medical history elements, were the primary drivers of STI risk prediction, with different ML algorithms emphasizing different aspects of these multifaceted relationships. Although SHAP substantially enhances model interpretability by quantifying feature-level contributions, ensemble learning approaches such as random forest and XGBoost remain inherently more complex than conventional statistical models. Therefore, future work should explore additional explainable AI methodologies and clinically interpretable frameworks to further strengthen transparency and support implementation in real-world healthcare settings.

### 3.5 Model performance

Analyzing the performance metrics across seven ML models (logistic regression, SGD classifier, decision tree, random forest, KNN, XGBoost, and MLP) for predicting STI risk, XGBoost emerged as the most effective solution, with random forest as a strong second. XGBoost demonstrated strong performance across all key evaluation metrics, achieving the highest accuracy (89.1%) and an excellent Kappa statistic (0.845), reflecting a high level of agreement beyond chance. The model exhibited outstanding recall (0.891) and precision (0.867), resulting in the highest F1-score (0.879) among all models, while maintaining the lowest RMSE (0.351) and RAE (11.7%). These balanced metrics are critical for healthcare screening, where both false negatives (missing at-risk individuals) and false positives can have serious consequences. XGBoost also achieved the highest ROC area (0.935), indicating strong discriminative ability across all classification thresholds. This superior ability of ensemble methods to identify at-risk cases while maintaining overall high accuracy aligns with Shahmoradi et al. (2021), who emphasized the importance of balanced sensitivity metrics in healthcare settings. Ensemble approaches (XGBoost and random forest) clearly outperformed simpler models, underscoring their value in capturing complex, non-linear relationships among STI risk factors. These findings are further supported by our recent published study and a previous systematic and meta-analysis review, demonstrated that ensemble learning approaches, particularly XGBoost-based models, consistently achieve superior predictive performance in HIV and STI risk assessment while highlighting the importance of external validation and implementation-focused studies for broader clinical translation (Ghosh et al., 2025; Latt et al., 2025). These findings complement Comulada et al., who reported the limitations of linear models, and support Mahajan et al. (Comulada et al., 2023; Mahajan et al., 2023), who highlighted the applicability of ensemble learning to infectious disease prediction.

Random forest performed strongly as the second-best model with an accuracy of 87.6% and a Kappa statistic of 0.821. It demonstrated excellent recall (0.876) and precision (0.843), yielding an impressive F1-score (0.859) and ROC area (0.921). The model showed good generalization with training accuracy of 89.7% compared to its test accuracy, indicating minimal overfitting. The decision tree also proved effective with an accuracy of 83.1% and a Kappa of 0.756, offering strong interpretability while maintaining good recall (0.831) and precision (0.793), with an F1-score of 0.812, providing interpretable results consistent with O’Sullivan’s recommendations (O’Sullivan, 2024).

The MLP model demonstrated strong performance with an accuracy of 85.2% and Kappa of 0.798, demonstrating good recall (0.852) and precision (0.821) with an F1-score of 0.836. The linear models, logistic regression and SGD classifier, showed similar moderate performance with accuracies of 82.5 and 82.3%, respectively. Both achieved Kappa values around 0.74 and F1-scores above 0.80. KNN showed the lowest performance among all models with an accuracy of 79.8%, Kappa of 0.703, and F1-score of 0.780, along with the highest RMSE (0.438) and RAE (18.1%). These results highlight the importance of selecting models that can accommodate complex interactions inherent in healthcare prediction tasks.

The feature importance analysis across models reveals that comorbidity (FH) was consistently ranked high, particularly in decision tree, random forest, and XGBoost models, while hygiene practice and behavioral factors show markedly influence in logistic regression and SGD models. XGBoost’s superior performance could be attributed to its ability to effectively capture complex interactions between medical factors and behavioral aspects through gradient boosting and advanced regularization techniques. SHAP analysis confirmed the key role of comorbidity (FH) in shaping model predictions across multiple algorithms, with education level and RTI medication identified as the next most influential predictors. Given these considerations, XGBoost emerges as the most clinically valuable model, offering the best balance of sensitivity, specificity, interpretability, and operational feasibility-key priorities in minimizing both missed diagnoses and false positives in STI screening applications. A comparison of model performance on the full predictor set versus a reduced set excluding symptom-related predictors is provided in Supplementary Table 3. Although the present models were developed using data collected from urban college-going women in Delhi, the underlying machine learning framework remains adaptable to other populations and settings. The workflow involving data preprocessing, feature engineering, model optimization, and SHAP-based interpretation can be readily applied to independent datasets. However, model recalibration and retraining using region-specific demographic, behavioral, and epidemiological data will be necessary before broader implementation to ensure robust predictive performance across diverse populations.

## 4 Conclusion

This study highlights the potential of machine learning to predict RTI risk stratification, screening support, and hypothesis generation with reasonable accuracy and interpretability. XGBoost proved to be the most effective algorithm, achieving the highest accuracy and F1-score, while Random Forest also performed strongly. SHAP analysis demonstrated the substantial influence of comorbidity, education, and RTI medication history, reinforcing the importance of integrating behavioral, clinical, and demographic factors in STI risk assessment. Future work should incorporate real-time patient data to enable dynamic risk prediction and expand these ML approaches to other infectious diseases such as to HIV. By supporting proactive, data-driven screening and targeted interventions, such models have the potential to strengthen public health strategies and improve population-level outcomes. While the present findings were generated from a specific urban academic population, the proposed explainable AI framework provides a scalable foundation that can be adapted and externally validated in broader community, clinical, and geographically diverse populations in future studies. In a cohort of 1,920 urban females in Delhi, ensemble models-particularly XGBoost-captured complex, nonlinear relationships across 30 demographic, reproductive, behavioural, and clinical variables. SHAP-based interpretability confirmed the importance of family history (FH), education, and prior RTI history as key risk determinants. These insights support practical use of ML models as pre-screening tools in college and community settings to prioritize clinical evaluation and health education among younger generations. However, models developed using self-reported survey data should be regarded as complementary to, rather than replacements for, clinical and laboratory diagnoses.

## 5 Limitations and future directions

Several limitations affect generalizability. First, the dataset comprises self-selected urban females from colleges of University of Delhi, Delhi, findings therefore reflect this specific cohort (students, faculty and staff) rather than wider populations in Delhi or India; in particular, rural women and women with lower educational attainment are not represented, and the findings should not be generalized to the broader Indian female population. Second, reliance on self-reported symptoms and history may introduce reporting bias and misclassification. Third, although SMOTE and careful pre-processing were applied to mitigate class imbalance and missingness, model performance depends on data quality, some predictors such as socio-economic status or microbiological confirmation were not available and could refine future models. Finally, ensemble models, while performant, are less transparent than simple linear models, SHAP helps but does not fully remove complexity.

Future work should validate these models in independent, externally sampled cohorts (including clinical/laboratory confirmed outcomes), incorporate additional socioeconomic and clinical biomarkers, and evaluate prospective or real-time risk prediction with integration into screening programs. Overall, our results support the role of interpretable ML as a useful adjunct for early RTI/STI risk identification in urban female populations and for guiding targeted public health interventions. Importantly, these models are intended to complement existing clinical practices by facilitating early risk stratification and should not be considered replacements for clinical examination, microbiological testing, or laboratory-confirmed diagnosis.

## Data Availability

The original contributions presented in the study are included in the article/Supplementary material, further inquiries can be directed to the corresponding author.
