# Supplementary material for: Prediction of female reproductive tract infections risk among college-going young adult women in Delhi using explainable artificial intelligence
Source: Front Artif Intell. 2026 Jul 17;9:1803913. doi: 10.3389/frai.2026.1803913 (PMC13424196; doi:10.3389/frai.2026.1803913)
Supplement: Supplementary file 2 [file Supplementary_file_2.docx]

**Supplementary Materials 2:**

**1. Application of Different Models:**

1. **K-Nearest Neighbors (KNN):** KNN is a non-parametric, proximity-based classification algorithm well-suited for capturing local data patterns. Its ability to handle nonlinear relationships makes it a relevant choice for predicting complex STI risk factors.
2. **Decision Tree:** Decision Trees provide high interpretability, allowing the model to uncover crucial interactions between symptoms, family history, and other variables contributing to STI risk. Their hierarchical structure is well-suited for this task.
3. **Random Forest:** As an ensemble method, Random Forest leverages the strengths of multiple Decision Trees to improve predictive performance and robustness. Its ability to capture nonlinearities and handle a variety of data types makes it a promising candidate.
4. **Stochastic Gradient Descent (SGD):** SGD is a computationally efficient algorithm, making it suitable for large-scale datasets. While its performance was weaker in this case, it was included to evaluate its suitability for STI risk prediction.
5. **Logistic Regression:** As a standard linear probability model, Logistic Regression provides insights into the directional relationships between predictors and STI risk, complementing the nonlinear approaches.
6. **Advanced ML Approach:**
7. **XGBoost Methodology**

XGBoost builds an ensemble of weak learners (typically decision trees) through gradient boosting. The model iteratively adds trees that correct errors made by previous trees. Each new tree aims to minimize a loss function using second-order gradients. Key methodological components include:

- Split finding through approximate greedy algorithm
- Handling missing values through default directions in tree nodes
- Built-in L1 (Lasso) and L2 (Ridge) regularization to prevent overfitting
- Column block for parallel learning

The model optimizes a regularized objective function that balances prediction accuracy with model complexity, using gradient and hessian information for optimal tree construction. The selection of these diverse models allowed us a careful and detailed evaluation of the various approaches and their ability to capture the complex factors associated with STI risk among the target population.


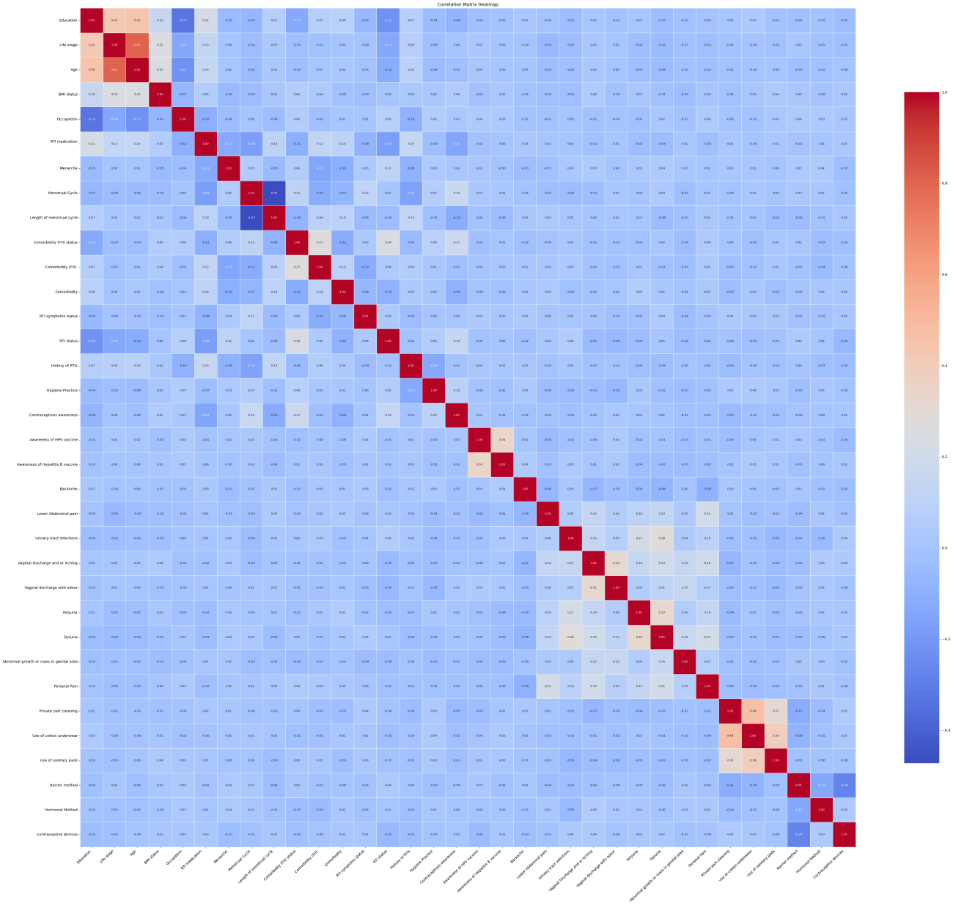


**Supplementary Figure 1:** Heatmap displaying the correlation coefficients among key risk factors for sexually transmitted infections (STIs), highlighting the strength and direction of their relationship. The diagonal red squares represent perfect correlation of each variable with itself (value of 1.0), while the varying shades of red, white, and blue across the rest of the matrix indicate positive correlations (red), no correlation (white), and negative correlations (blue) between different pairs of variables.

**Supplementary Table 1: Percentage of Missing Data Per Variable**

| **S.No.** | **Variable** | **Missing Values (n)** | **Missing (%)** |
| --- | --- | --- | --- |
| 1 | Age | 12 | 0.62 |
| 2 | BMI Status | 18 | 0.94 |
| 3 | Education | 7 | 0.36 |
| 4 | Life Stage | 9 | 0.47 |
| 5 | Occupation | 5 | 0.26 |
| 6 | RTI Medication | 11 | 0.57 |
| 7 | Menarche | 21 | 1.09 |
| 8 | Menstrual Cycle | 8 | 0.42 |
| 9 | Length of Menstrual Cycle | 14 | 0.73 |
| 10 | Comorbidity (Family History) | 16 | 0.83 |
| 11 | RTI Symptoms Status | 6 | 0.31 |
| 12 | History of RTIs | 9 | 0.47 |
| 13 | Hygiene Practice | 4 | 0.21 |
| 14 | Contraceptives Awareness | 10 | 0.52 |
| 15 | Backache | 3 | 0.16 |
| 16 | Lower Abdominal Pain | 5 | 0.26 |
| 17 | Urinary Tract Infections | 7 | 0.36 |
| 18 | Vaginal Discharge and/or Itching | 8 | 0.42 |
| 19 | Vaginal Discharge with Odour | 6 | 0.31 |
| 20 | Polyuria | 4 | 0.21 |
| 21 | Dysuria | 5 | 0.26 |
| 22 | Abnormal Growth or Mass in Genital Area | 3 | 0.16 |
| 23 | Perianal Pain | 4 | 0.21 |
| 24 | Private Part Cleaning | 2 | 0.10 |
| 25 | Use of Cotton Underwear | 3 | 0.16 |
| 26 | Use of Sanitary Pads | 4 | 0.21 |
| 27 | Barrier Method | 7 | 0.36 |
| 28 | Hormonal Method | 6 | 0.31 |
| 29 | Contraceptive Devices | 8 | 0.42 |
| 30 | RTI Status (History of Diagnosis) | 10 | 0.52 |
|  | **Overall (any variable)** | **≤5% per variable** | **All <2.0** |

*Note: Missing values were imputed using mean substitution (continuous variables) or mode (categorical variables). Sensitivity analyses comparing imputed and complete-case results yielded comparable model performance across all ML algorithms.*

**Supplementary Table 2: Hyperparameter Ranges Tested and Selected Values for Each ML Model**

| **Model** | **Hyperparameter** | **Range Tested** | **Selected Value** |
| --- | --- | --- | --- |
| Decision Tree | Criterion | {gini, entropy} | entropy |
|  | Max depth | 3, 5, 7, 10, None | 7 |
|  | Min samples split | 2, 5, 10, 20 | 5 |
|  | Min samples leaf | 1, 2, 4, 8 | 2 |
| Random Forest | n_estimators | 50, 100, 200, 300 | 200 |
|  | Max depth | 5, 10, 15, None | 10 |
|  | Min samples split | 2, 5, 10 | 2 |
|  | Min samples leaf | 1, 2, 4 | 1 |
| KNN | n_neighbors | 3, 5, 7, 9, 11, 15 | 7 |
|  | Metric | {euclidean, manhattan} | manhattan |
|  | Weights | {uniform, distance} | distance |
| XGBoost | n_estimators | 50, 100, 200, 500 | 200 |
|  | Max depth | 3, 5, 7, 9 | 6 |
|  | Learning rate | 0.01, 0.05, 0.1, 0.2, 0.3 | 0.1 |
|  | Subsample | 0.6, 0.8, 1.0 | 0.8 |

*Note: All models used random_state = 42. Train-test split: 80:20. Hyperparameter optimization via 5-fold cross-validation using grid search and randomized search. SMOTE applied to the training set only.*

**Supplementary Table 3: Model Performance Comparison-Full Predictor Set vs. Reduced Set (Excluding Symptom-Related Predictors)**

| **Model** | **Accuracy (Full)** | **Accuracy (Reduced)** | **ROC-AUC (Full)** | **ROC-AUC (Reduced)** | **F1 (Full)** | **F1 (Reduced)** |
| --- | --- | --- | --- | --- | --- | --- |
| Logistic Regression | 82.5% | 76.3% | 0.871 | 0.812 | 0.807 | 0.748 |
| SGD Classifier | 82.3% | 75.8% | 0.869 | 0.808 | 0.804 | 0.746 |
| Decision Tree | 83.1% | 77.4% | 0.878 | 0.819 | 0.812 | 0.753 |
| Random Forest | 87.6% | 81.2% | 0.921 | 0.864 | 0.859 | 0.801 |
| KNN | 79.8% | 73.1% | 0.851 | 0.793 | 0.780 | 0.721 |
| XGBoost | 89.1% | 83.5% | 0.935 | 0.881 | 0.879 | 0.824 |
| MLP | 85.2% | 79.6% | 0.898 | 0.843 | 0.836 | 0.779 |

*Note: “Full” = all 30 predictors including symptom-related variables (vaginal discharge, dysuria, lower abdominal pain, RTI symptoms status, history of RTI, RTI medication). “Reduced” = symptom-related predictors excluded. The moderate performance drop in the reduced set confirms that these variables contribute meaningfully but that data leakage is limited, as performance remains in a plausible range without them.*
